# Perceived barriers to early presentation and symptom-specific time to seek medical advice for possible colorectal cancer symptoms among Palestinians

**DOI:** 10.1038/s41598-023-34136-5

**Published:** 2023-04-27

**Authors:** Mohamedraed Elshami, Mohammed Ayyad, Fatma Khader Hamdan, Mohammed Alser, Ibrahim Al-Slaibi, Shoruq Ahmed Naji, Balqees Mustafa Mohamad, Wejdan Sudki Isleem, Adela Shurrab, Bashar Yaghi, Yahya Ayyash Qabaja, Mohammad Fuad Dwikat, Raneen Raed Sweity, Remah Tayseer Jneed, Khayria Ali Assaf, Maram Elena Albandak, Mohammed Madhat Hmaid, Iyas Imad Awwad, Belal Khalil Alhabil, Marah Naser Alarda, Amani Saleh Alsattari, Moumen Sameer Aboyousef, Omar Abdallah Aljbour, Rinad AlSharif, Christy Teddy Giacaman, Ali Younis Alnaga, Ranin Mufid Abu Nemer, Nada Mahmoud Almadhoun, Sondos Mahmoud Skaik, Nasser Abu-El-Noor, Bettina Bottcher

**Affiliations:** 1 Division of Surgical Oncology, Department of Surgery, University Hospitals Cleveland Medical Center, 11100 Euclid Avenue, Lakeside 7100, Cleveland, OH 44106 USA; 2 Ministry of Health, Gaza, Palestine; 3 Faculty of Medicine, Al-Quds University, Jerusalem, Palestine; 4 The United Nations Relief and Works Agency for Palestine Refugees in The Near East (UNRWA), Amman, Jordan; 5 Almakassed Hospital, Jerusalem, Palestine; 6 Faculty of Pharmacy, Al-Azhar University of Gaza, Gaza, Palestine; 7 Doctors Without Borders (Médecins Sans Frontières), Hebron, Palestine; 8 Faculty of Medicine, Islamic University of Gaza, Gaza, Palestine; 9 Palestine Medical Complex, Khanyounis, Palestine; 10 Faculty of Medicine, An-Najah National University, Nablus, Palestine; 11 Faculty of Dentistry, Arab American University, Jenin, Palestine; 12 Augusta Victoria Hospital, Jerusalem, Palestine; 13 Faculty of Allied Medical Sciences, Arab American University, Jenin, Palestine; 14 Faculty of Medicine, Al-Azhar University, Gaza, Palestine; 15 Faculty of Medicine, Al-Quds Abu Dis University Al-Azhar Branch of Gaza, Gaza, Palestine; 16 Faculty of Nursing, Islamic University of Gaza, Gaza, Palestine

**Keywords:** Cancer, Signs and symptoms

## Abstract

This study explored the anticipated time to seek medical advice for possible colorectal cancer (CRC) signs/symptoms and its association with CRC symptom awareness. In addition, it examined perceived barriers that may delay seeking medical advice. Palestinian adults were recruited from hospitals, primary healthcare centers, and public spaces in 11 governorates. A modified, translated-into-Arabic version of the validated Bowel Cancer Awareness Measure was used. The questionnaire comprised three sections: sociodemographics, assessment of CRC symptom awareness and time to seek medical advice, and barriers to early presentation. A total of 4623 participants were included. The proportion that reported seeking immediate medical advice for possible CRC signs/symptoms with blood or mass ranged from 47.1% for ‘blood in stools’ to 59.5% for ‘bleeding from back passage’. Less than half of the participants reported immediate seeking of medical advice for non-specific symptoms (ranging from 5.4% for ‘loss of appetite’ to 42.0% for ‘anemia’) and other gastrointestinal symptoms (ranging from 7.7% for ‘feeling persistently full’ to 35.7% for ‘change in bowel habits’). Good CRC symptom awareness was associated with higher likelihood of seeking medical advice within a week from recognizing a CRC symptom. About 13.0% reported a delay to visit their doctor after recognizing a CRC symptom. The most reported barriers were practical with ‘would try some herbs first’ (50.9%) as the leading barrier. CRC symptoms with blood or mass prompted earlier help seeking. Participants with good CRC awareness were more likely to seek medical advice within a week.

## Introduction

Globally, colorectal cancer (CRC) is the third most common cancer accounting for 10.0% of the total cancer cases and the second most common cause of cancer-related mortality; accounting for 9.4% of total cancer deaths^1^. In Western Asia, a total of 41,569 cases of CRC were registered in 2020^2^. In Palestine, CRC is the most common cancer among males and the second most common cancer among females after breast cancer^3^. CRC accounted for 13.7% of the total cancer cases in the West Bank and Jerusalem (WBJ) in 2021 with an incidence rate of 16.4 per 100,000 general population, and 12.8% of the total cancer cases in the Gaza Strip with an incidence rate of 11.5 per 100,000 general population^3,4^.

Previous studies have shown an association between the nature of CRC signs/symptoms and the time needed to seek medical advice^5,6^. Esteva and colleagues from Spain found that patients, who presented with abdominal pain or vomiting, had a shorter time to seek medical advice than those with other signs/symptoms. The authors also found that a patient’s perception of the seriousness of a sign/symptom led to shorter intervals to seeking medical advice and early diagnosis^5^.

Early diagnosis plays an important role in improving the prognosis and survival rates of CRC^7^. Immediate seeking of medical advice is a key to early diagnosis, but there are several factors that can delay seeking medical advice. These include the ability to recognize CRC signs/symptoms and the existence of barriers to early presentation, which could be emotional, practical or service-related barriers^8^. A recent national study from Palestine has demonstrated that only 40% of the participants displayed good awareness of CRC signs/symptoms^9^. Another study conducted in the Gaza Strip found that the most common emotional barrier encountered by participants was ‘feeling worried about what the doctor might find’^8^. However, the interplay between recognition of CRC signs/symptoms with the time to seek medical advice as well as having barriers to early presentation warrants further investigation.

Therefore, this national study aimed to: (i) explore the anticipated time to seek medical advice for a possible CRC sign/symptom, (ii) examine the association between CRC signs/symptom awareness and the time to seek medical advice, and (iii) examine the barriers that may delay help-seeking and their association with the awareness levels of CRC signs/symptoms.

## Materials and methods

### Study design and population

This was a national cross-sectional study, where data were collected between the 1st of July 2019 and the 31st of March 2020. The target population was adult (≥ 18 years) Palestinians, who make up 62.2% of about five million living in the Gaza Strip and the WBJ^10^. Palestine has 16 governorates: five in the Gaza Strip and 11 in the WBJ^10^. Participants were recruited from 11 governorates: four in the Gaza Strip and seven in the WBJ.

### Settings and sampling methods

In Palestine, healthcare is provided by governmental facilities, non-governmental organizations (NGOs), private healthcare organizations, and the United Nations Relief and Works Agency (UNRWA) facilities. The majority of patients utilize health services provided by governmental hospitals or primary healthcare centers at no cost or little co-payments^3^. To maximize the representativeness of the study cohort, potential participants were invited to participate from governmental hospitals, primary healthcare centers, and public spaces, such as mosques, churches, downtown areas, parks, trade streets, malls and public transportation stations using convenience sampling.

### Inclusion and exclusion criteria

Inclusion criteria for this study were being an adult (≥ 18 years) Palestinian and being a visitor to one of the data collection sites. Exclusion criteria included working or studying in a health-related field, holding a nationality other than Palestinian, being unable to complete the questionnaire, and being a visitor to an oncology department at the time of data collection.

### Data collection and measurement tool

A modified version of the validated tool Bowel Cancer Awareness Measure (BoCAM) was used for data collection^11^. BoCAM was translated into Arabic and then back translated into English by four bilingual healthcare professionals (two at each stage) who had experience in clinical research and survey design. To ensure content validity, the translated questionnaire was reviewed by five independent experts specialized in the fields of coloproctology, gastroenterology, and public health. This was then followed by conducting a pilot study (n = 25) to assess the clarity of the questions of the Arabic version. The data collected in the pilot study were not included in the final data analysis. Internal consistency was assessed using Cronbach’s Alpha, which reached an acceptable value (α = 0.887).

The questionnaire comprised three sections. The first section described participants’ socio-demographic factors including age, gender, marital status, level of education, employment status, monthly income, place of residence, having a chronic health condition, following a vegetarian diet, and knowing someone with cancer. The second section evaluated the participants’ awareness of 12 CRC signs and symptoms based on a 5-point Likert scale (1 = strongly disagree, 2 = disagree, 3 = not sure, 4 = agree, and 5 = strongly agree). Out of the 12 CRC signs/symptoms, nine were adapted from the original BoCAM, where they had yes/no/unknown responses but were then converted into a 5-point Likert scale questions to minimize the possibility of answering randomly^8,9,12–20^. The signs/symptoms of ‘feeling persistently full’, ‘unexplained loss of appetite’, and ‘unexplained generalized fatigue’ were added to the questionnaire since they were used in other forms of the Cancer Awareness Measure^21–23^. The second section also assessed the anticipated time to seek medical advice for each possible CRC sign/symptom when recognized using an open-ended question. The third section evaluated perceived barriers to early presentation based on the aforementioned 5-point Likert scale. A total of 10 barriers were adopted from the original BoCAM^22^, whereas the remaining were deemed important to include based on previous similar studies^24–26^.

Participants were invited to complete the questionnaire in a face-to-face interview with the presence of a data collector. The data were collected using ‘Kobo Toolbox’, a secure, user-friendly data collection tool that can be easily accessed via smartphones^27^.

### Statistical analysis

The American Cancer Society recommends starting CRC screening for people at average risk at the age of 45^28^. Therefore, the continuous variable of age was categorized into two categories using this cutoff: 18–44 years and ≥ 45 years. The minimum wage in Palestine is 1450 NIS (about $450) ^29^. Therefore, monthly income was categorized using this cutoff into two categories: < 1450 NIS and ≥ 1450 NIS.

Median and interquartile ranges (IQR) were used to describe non-normally distributed, continuous variables, whereas frequencies and percentages were used to describe categorical variables. Comparisons between baseline characteristics of participants recruited from the Gaza Strip and those recruited from the WBJ were performed using the Kruskal–Wallis test if the characteristic was continuous or the Pearson’s Chi-square test if it was categorical.

CRC signs/symptoms were categorized into three categories: (i) signs/symptoms with a mass or blood, (ii) signs/symptoms of a non-specific nature, and (iii) other gastrointestinal signs/symptoms. The anticipated time to seek medical advice for a possible CRC sign/symptom was categorized into four categories: immediately (less than 24 h), 2–7 days, > 7 days, and never. Frequencies and percentages were used to describe the anticipated time and comparisons between participants recruited from the Gaza Strip and those recruited from the WBJ were performed using the Pearson’s Chi-Square test. The awareness level of CRC signs and symptoms was evaluated as previously described^9,12–17^. Answers provided by participants who responded with agree or strongly agree were considered correct, and other answers were considered incorrect. One point was given for each correctly recognized CRC sign/symptom. The total score was calculated (ranging from 0 to 12) and categorized, based on the number of CRC signs/symptoms recognized, into three categories: poor (0–4), fair (5–8), and good awareness (9–12). Multivariable logistic regression was utilized to examine the association between awareness level of CRC signs/symptoms and the anticipated time to seek medical advice for a possible CRC sign or symptom. The multivariable analysis adjusted for age group, gender, education, employment status, monthly income, place of residency, marital status, having a chronic disease, following a vegetarian diet, knowing someone with cancer, and site of data collection. This model was determined a priori based on previous studies^6,8,9,12,24–26^.

Consistent with the original BoCAM^22^, barriers to early presentation were categorized into three categories: emotional, service, and practical barriers. Frequencies and percentages were used to describe the displayed barriers and comparisons between participants recruited from the Gaza Strip and those recruited from the WBJ were performed using Pearson’s Chi-Square test. The median number of barriers in each category (overall, emotional, service, and practical) was utilized as a cutoff to dichotomize the number of displayed barriers. This was followed by running a multivariable logistic regression to examine the association between CRC signs/symptom awareness level and displaying at least the median number of barriers in each category. The same aforementioned multivariable model was used in all those analyses.

Missing data were hypothesized to occur completely at random and were handled using a complete case analysis. Data were analyzed using Stata software version 16.0 (StataCorp, College Station, Texas, United States).

### Ethical approval and consent to participate

Prior to data collection, ethical approval had been sought and obtained from the Research Ethics Committee at the Islamic University of Gaza, the Human Resources Development department at the Palestinian Ministry of Health, and the Helsinki Committee in the Gaza Strip. The study along with its purpose and objectives were thoroughly explained to the participants and they were well informed that their participation is completely voluntary. A written informed consent was obtained from each participant prior to filling out the questionnaire, and data were collected and reported anonymously. All methods were performed in accordance with the relevant guidelines and regulations.

## Results

### Participant characteristics

Of the 5254 participants approached, 4877 participants agreed and completed the questionnaire (response rate = 92.3%). A total of 4623 questionnaires were included in the final analysis (254 were excluded: 210 had missing data and 44 did not meet the inclusion criteria); 2700 were from the WBJ and 1923 from the Gaza Strip. The median age [IQR] among all study participants was 31.0 [24.0, 43.0] years and 1879 (40.6%) were males (Table 1). Participants from the Gaza Strip were younger, had lower monthly income, and suffered from a fewer number of chronic diseases than participants from the WBJ. The majority of participants displayed fair (42%) to good (40%) levels of awareness about CRC signs/symptoms. Participants from the WBJ were more likely than participants from the Gaza Strip to display good awareness of CRC signs/symptoms (42.1% vs. 37.0%).

**Table 1:** Characteristics of study participants.

Characteristic	Total (n = 4623)	Gaza strip (n = 1923)	WBJ (n = 2700)	*p*–value
Age, median [IQR]	31.0 [24.0, 43.0]	30.0 [24.0, 40.0]	32.0 [24.0, 44.0]	< 0.001*
Age group, n (%)				
18 to 44	3608 (78.1)	1579 (82.1)	2029 (75.1)	< 0.001
45 or older	1015 (21.9)	344 (17.9)	671 (24.9)	
Gender, n (%)				
Male	1879 (40.6)	710 (36.9)	1169 (43.3)	< 0.001
Female	2744(59.4)	1213(63.1)	1531(56.7)	
Educational level, n (%)				
Secondary or below	2217 (47.9)	946 (49.2)	1271 (47.1)	0.16
Post–secondary	2406 (52.1)	977 (50.8)	1429 (52.9)	
Occupation, n (%)				
Unemployed/housewife	2067 (44.7)	1112 (57.8)	955 (35.4)	< 0.001
Employed	1898 (41.1)	563 (29.3)	1335 (49.4)	
Retired	96 (2.1)	29 (1.5)	67 (2.5)	
Student	562 (12.1)	219 (11.4)	343 (12.7)	
Monthly income ≥ 1450 NIS, n (%)	3039 (65.7)	559 (29.1)	2480 (91.9)	< 0.001
Marital status, n (%)				
Single	1414 (30.5)	548 (28.5)	866 (32.1)	0.032
Married	3067 (66.4)	1316 (68.4)	1751 (64.8)	
Divorced/Widowed	142 (3.1)	59 (3.1)	83 (3.1)	
Having a chronic disease, n (%)	906 (19.6)	314 (16.3)	592 (21.9)	< 0.001
Following a vegetarian diet, n (%)	560 (12.1)	386 (20.1)	174 (6.4)	< 0.001
Knowing someone with cancer, n (%)	2395 (51.8)	1007 (52.4)	1388 (51.4)	0.52
Site of data collection, n (%)				
Public Spaces	1450 (31.4)	491 (25.5)	959 (35.5)	< 0.001
Hospitals	1659 (35.9)	690 (35.9)	969 (35.9)	
Primary healthcare centers	1514 (33.7)	742 (38.6)	772 (28.6)	
Awareness of CRC symptoms, n (%)				
Poor	833 (18.0)	365 (19.0)	468 (17.3)	0.002
Fair	1941 (42.0)	847 (44.0)	1094 (40.5)	
Good	1849 (40.0)	711 (37.0)	1138 (42.1)	

n = number of participants, IQR = interquartile range, WBJ = West Bank and Jerusalem.

All *p*-values are for Pearson’s Chi-square test.

### Immediate seeking of medical advice for a possible CRC sign/symptom

The proportion of study participants who reported that they would immediately seek medical advice for possible CRC signs/symptoms with blood or a mass was 59.5% (n = 2752) for ‘bleeding from back passage’, 52.9% (n = 2445) for ‘lump in the abdomen’, and 47.1% (n = 2176) for ‘blood in stools’ (Table 2). Participants from the WBJ were more likely than participants from the Gaza Strip to immediately visit a doctor if they had “blood in the stool,” but were less likely to immediately seek medical advice if they experienced a “lump in the abdomen”.

**Table 2:** Summary of the reported time to seek medical advice for a possible colorectal cancer symptom.

Category of signs/symptoms	CRC sign/symptom	Total (n = 4623) n (%)	Gaza strip (n = 1923) n (%)	WBJ (n = 2700) n (%)	*p*-value
Signs/symptoms with a mass or blood	Bleeding from back passage				
	Immediately	2752 (59.5)	1136 (59.1)	1616 (59.9)	
	≤ 7 days	1601 (34.6)	682 (35.5)	919 (34.0)	0.59
	> 7 days	52 (1.2)	22 (1.1)	30 (1.1)	
	Never	218 (4.7)	83 (4.3)	135 (5.0)	
	Lump in the abdomen				
	Immediately	2445 (52.9)	1059 (55.1)	1386 (51.3)	
	≤ 7 days	178 (38.6)	717 (37.3)	1068 (39.6)	0.002
	> 7 days	134 (2.9)	63 (3.3)	71 (2.6)	
	Never	259 (5.6)	84 (4.3)	175 (6.5)	
	Blood in the stools				
	Immediately	2176 (47.1)	857 (44.6)	1319 (48.9)	
	≤ 7 days	1958 (42.4)	869 (45.2)	1089 (40.3)	0.011
	> 7 days	76 (1.6)	32 (1.7)	44 (1.6)	
	Never	413 (8.9)	165 (8.6)	248 (9.2)	
Signs/symptoms of a non-specific nature	Anemia				
	Immediately	1943 (42.0)	750 (39.0)	1193 (44.2)	
	≤ 7 days	1631 (35.3)	767 (39.9)	864 (32.0)	< 0.001
	> 7 days	300 (6.5)	107 (5.6)	193 (7.1)	
	Never	749 (16.2)	299 (15.5)	450 (16.7)	
	Unexplained weight loss				
	Immediately	974 (21.1)	377 (19.6)	597 (22.1)	
	≤ 7 days	1437 (31.1)	744 (38.7)	693 (25.7)	< 0.001
	> 7 days	496 (10.7)	193 (10.0)	303 (11.2)	
	Never	1716 (37.1)	609 (31.7)	1107 (41.0)	
	Unexplained generalized fatigue immediately				
	≤ 7 days	313 (6.8)	103 (5.4)	210 (7.8)	
	> 7 days	1610 (34.8)	832 (43.3)	778 (28.8)	< 0.001
	Never	359 (7.8)	116 (6.0)	243 (9.0)	
		2341 (50.6)	872 (45.3)	1469 (54.4)	
	Unexplained loss of appetite				
	Immediately	249 (5.4)	58 (3.0)	191 (7.1)	
	≤ 7 days	1307 (28.3)	742 (38.6)	565 (20.9)	< 0.001
	> 7 days	346 (7.5)	162 (8.4)	184 (6.8)	
	Never	2721 (58.8)	961 (50.0)	1760 (65.2)	
Other gastrointestinal signs/symptoms	Change in bowel habits				
	Immediately	1650 (35.7)	549 (28.5)	11.1 (40.8)	
	≤ 7 days	1905 (41.2)	973 (50.6)	932 (34.5)	< 0.001
	> 7 days	202 (4.4)	72 (3.7)	130 (4.8)	
	Never	866 (18.7)	329 (17.1)	537 (19.9)	
	Persistent pain in the abdomen Immediately				
	≤ 7 days	1086 (23.5)	410 (21.3)	676 (25.0)	
	> 7 days	2682 (58.0)	1172 (60.9)	1510 (55.9)	0.002
	Never	101 (2.2)	48 (2.5)	53 (2.0)	
		754 (16.3)	293 (15.3)	461 (17.1)	
	Pain in the back passage				
	Immediately	977 (21.1)	335 (17.4)	642 (23.8)	
	≤ 7 days	2308 (49.9)	1095 (56.9)	1213 (44.9)	< 0.001
	> 7 days	141 (3.1)	67 (3.5)	74 (2.7)	
	Never	1197 (25.9)	426 (22.2)	771 (28.6)	
	Bowel does not completely empty after using the lavatory				
	Immediately				
	≤ 7 days	436 (9.4)	151 (7.9)	285 (10.6)	< 0.001
	> 7 days	2062 (44.6)	1011 (52.6)	1051 (38.9)	
	Never	202 (4.4)	99 (5.1)	103 (3.8)	
		1923 (41.6)	662 (34.4)	1261 (46.7)	
	Feeling persistently full				
	Immediately	357 (7.7)	143 (7.4)	214 (7.9)	
	≤ 7 days	1994 (43.1)	1053 (54.8)	941 (34.9)	< 0.001
	> 7 days	295 (6.4)	122 (6.3)	173 (6.4)	
	Never	1977 (42.8)	605 (31.5)	1372 (50.8)	

n = number of participants, WBJ = West Bank and Jerusalem.

All *p*-values are for Pearson’s Chi-square test.

The proportion of study participants who reported that they would immediately seek medical advice for a possible non-specific CRC sign/symptom was 42.0% (n = 1943) for ‘anemia’, 21.1% (n = 974) for ‘unexplained weight loss’, 6.8% (n = 313) for ‘unexplained generalized fatigue’, and 5.4% (n = 249) for ‘unexplained loss of appetite’. Participants from the WBJ were more likely than participants from the Gaza Strip to immediately seek medical advice when asked about all non-specific CRC signs/symptoms.

The proportion of study participants who reported that they would immediately seek medical advice for other gastrointestinal signs/symptoms was 35.7% (n = 1650) for ‘change in bowel habits’, 23.5% (n = 1086) for ‘persistent pain in the abdomen, 21.1% (n = 977) for ‘pain in the back passage’, 9.4% (n = 436) for ‘bowel not emptying completely’, and 7.7% (n = 357) for ‘feeling persistently full’. Participants from the WBJ were more likely than participants from the Gaza Strip to immediately seek medical advice when asked about all other gastrointestinal signs/symptoms.

### Association between awareness level of CRC signs/symptoms and no seeking of medical advice

There was an associated decrease in the likelihood of reporting no seeking of medical advice for each of the CRC signs/symptoms among participants who displayed fair levels of awareness of CRC signs/symptoms compared to those who displayed poor levels (Table 3). Additionally, there was a further associated decrease in the likelihood of not seeking medical advice for all CRC signs/symptoms among participants who displayed good awareness of CRC signs/symptoms (Table 3).

**Table 3:** Multivariable logistic regression analyzing the association between no seeking of medical advice and awareness of colorectal cancer symptoms.

Awareness of CRC signs/symptoms	A) Signs/symptoms with a mass or blood									
	Lump in the abdomen		Blood in the stools		Bleeding from back passage					
	AOR (95% CI)	*p*-value	AOR (95% CI)	*p*-value	AOR (95% CI)	*p*-value				
Poor	Ref	Ref	Ref	Ref	Ref	Ref				
Fair	0.58 (0.42- 0.79)	0.001	0.53 (0.41- 0.69)	< 0.001	0.56 (0.40- 0.78)	0.001				
Good	0.43 (0.31- 0.61)	< 0.001	0.39 (0.30- 0.51)	< 0.001	0.37 (0.26- 0.54)	< 0.001				

Awareness of CRC signs/symptoms	B) Signs/symptoms of a non-specific nature									
	Unexplained weight loss		Unexplained generalized fatigue		Unexplained loss of appetite		Anemia			
	AOR (95% CI)	*p*-value	AOR (95% CI)	*p*-value	AOR (95% CI)	*p*-value	AOR (95% CI)	*p*-value		
Poor	Ref	Ref	Ref	Ref	Ref	Ref	Ref	Ref		
Fair	0.70 (0.59–0.83)	< 0.001	0.71 (0.59–0.84)	< 0.001	0.75 (0.63–0.90)	0.002	0.70 (0.57–0.86)	0.001		
Good	0.54 (0.45–0.64)	< 0.001	0.57 (0.47–0.67)	< 0.001	0.55 (0.46–0.66)	< 0.001	0.43 (0.35–0.54)	< 0.001		

Awareness of CRC signs/symptoms	C) Other gastrointestinal signs/symptoms									
	Feeling persistently full		Change in bowel habits		Persistent pain in the abdomen		Bowel does not completely empty after using the lavatory		Pain in the back passage	
	AOR (95% CI)	*p*-value	AOR (95% CI)	*p*-value	AOR (95% CI)	*p*-value	AOR (95% CI)	*p*-value	AOR (95% CI)	*p*-value
Poor	Ref	Ref	Ref	Ref	Ref	Ref	Ref	Ref	Ref	Ref
Fair	0.76 (0.64–0.91)	0.002	0.77 (0.63– 0.95)	0.012	0.73 (0.59– 0.89)	0.002	0.76 (0.65–0.91)	0.002	0.75 (0.63–0.91)	0.003
Good	0.61 (0.51–0.72)	< 0.001	0.56 (0.45–0.69)	< 0.001	0.43 (0.34–0.54)	< 0.001	0.55 (0.47–0.66)	< 0.001	0.52 (0.43–0.63)	< 0.001

AOR = adjusted odds ratio, CI = confidence interval, CRC = colorectal cancer.

All analyses were adjusted for age-group, gender, educational level, occupation, monthly income, having a chronic disease, following a vegetarian diet, knowing someone with cancer, marital status, residency, and site of data collection.

The outcome in all models is binary, where answers with ‘never’ were considered as ‘yes’ and all other answers were considered as ‘no’.

All p-values are for the multivariable logistic regression analysis.

### Association between awareness level of CRC signs/symptoms and seeking medical advice within a week

Compared to participants who displayed poor awareness of CRC signs/symptoms, participants with a fair level of awareness had an associated increase in the likelihood of seeking medical advice within a week for each of the CRC signs/symptoms except for ‘change in bowel habits’, where no associated difference was found (Table 4). Additionally, there was a further associated increase in the likelihood of seeking medical advice within a week for all CRC signs/symptoms among participants who displayed good awareness of CRC signs/symptoms.

**Table 4:** Multivariable logistic regression analyzing the association between seeking medical advice within a week and awareness of colorectal cancer symptoms.

Awareness of CRC signs/symptoms	A) Signs/symptoms with a mass or blood									
	Lump in the abdomen		Blood in the stools		Bleeding from back passage					
	AOR (95% CI)	*p*-value	AOR (95% CI)	*p*-value	AOR (95% CI)	*p*-value				
Poor	Ref	Ref	Ref	Ref	Ref	Ref				
Fair	1.46 (1.11–1.91)	0.007	1.56 (1.22–1.99)	< 0.001	1.60 (1.17–2.18)	0.003				
Good	2.00 (1.50–2.67)	< 0.001	2.14 (1.65–2.78)	< 0.001	2.16 (1.55–3.02)	< 0.001				

Awareness of CRC signs/symptoms	B) Signs/symptoms of a non-specific nature									
	Unexplained weight loss		Unexplained generalized fatigue		Unexplained loss of appetite		Anemia			
	AOR (95% CI)	*p*-value	AOR (95% CI)	*p*-value	AOR (95% CI)	*p*-value	AOR (95% CI)	*p*-value		
Poor	Ref	Ref	Ref	Ref	Ref	Ref	Ref	Ref		
Fair	1.40 (1.18–1.66)	< 0.001	1.38 (1.15–1.64)	< 0.001	1.24 (1.03–1.49)	0.022	1.32 (1.09–1.59)	0.004		
Good	1.55 (1.30- 1.84)	< 0.001	1.68 (1.41- 2.01)	< 0.001	1.61 (1.34- 1.95)	< 0.001	1.97 (1.61- 2.40)	< 0.001		

Awareness of CRC signs/symptoms	C) Other gastrointestinal signs/symptoms									
	Feeling persistently full		Change in bowel habits		Persistent pain in the abdomen		Bowel does not completely empty after using the lavatory		Pain in the back passage	
	AOR (95% CI)	*p*-value	AOR (95% CI)	*p*-value	AOR (95% CI)	*p*-value	AOR (95% CI)	*p*-value	AOR (95% CI)	*p*-value
Poor	Ref	Ref	Ref	Ref	Ref	Ref	Ref	Ref	Ref	Ref
Fair	1.24 (1.05–1.47)	0.014	1.71 (0.97–1.42)	0.11	1.38 (1.13–1.68)	0.001	1.26 (1.06–1.49)	0.008	1.28 (1.07– 1.54)	0.006
Good	1.45 (1.22–1.73)	< 0.001	1.53 (1.25–1.87)	< 0.001	2.07 (1.68–2.56)	< 0.001	1.65 (1.39–1.96)	< 0.001	1.70 (1.41–2.05)	< 0.001

AOR = adjusted odds ratio, CI = confidence interval, CRC = colorectal cancer.

All analyses were adjusted for age-group, gender, educational level, occupation, monthly income, having a chronic disease, following a vegetarian diet, knowing someone with cancer, marital status, residency, and site of data collection.

The outcome in all models is binary, where answers with ‘1–7 days’ were considered as ‘yes’ and all other answers were considered as ‘no’.

All *p*-values are for the multivariable logistic regression analysis.

### Barriers to early presentation and association with CRC symptom awareness

When participants were asked if they would delay seeking medical advice after recognizing a sign/symptom of CRC, 609 (12.5%) answered with ‘yes’ and reported at least one barrier to early presentation. Overall, the most commonly reported barriers were practical and those included ‘would try some herbs first’ (n = 310, 50.9%), ‘would think that symptom is not something important to see the doctor for’ (n = 305, 50.1%), and ‘too busy to go to the doctor’ (n = 304, 49.9%) (Table 5). The most commonly reported service-related barrier was ‘the thought that there is a small number of qualified doctors in the Palestinian Ministry of Health facilities’ (n = 260, 42.7%). The most commonly reported emotional barriers were ‘disliking the visit to healthcare facilities’ (n = 284, 46.6%) and ‘feeling worried about what a doctor might find’ (n = 258, 42.4%). All these findings were consistent in both the Gaza Strip and the WBJ.

**Table 5:** Summary of the reported barriers to early presentation among study participants.

Category	Barrier	Total (n = 609) n (%)	Gaza strip (n = 199) n (%)	WBJ (n = 410) n (%)	*p*-value
Emotional	Disliking the visit to healthcare facilities	284 (46.6)	94 (47.2)	190 (46.3)	0.86
	Feeling worried about what a doctor might find	258 (42.4)	86 (43.2)	172 (42.0)	0.79
	Feeling scared	245 (40.2)	71 (35.7)	174 (42.4)	0.11
	Would not trust the doctor	216 (35.5)	79 (39.7)	137 (33.4)	0.15
	Would prefer not to know the reason of the symptom	204 (33.5)	74 (37.2)	130 (31.7)	0.20
	God is the ultimate healer so there in no need to go to the doctor	200 (32.8)	74 (37.2)	126 (30.7)	0.12
	Would not feel comfortable to be examined by a doctor of a different gender from the participant’s	188 (30.9)	75 (37.7)	113 (27.6)	0.020
	Embarrassment	158 (25.9)	60 (30.2)	98 (23.9)	0.11
	Would not feel confident talking about symptom with	76 (12.5)	25 (12.6)	51 (12.4)	1.00
Service	The thought that there is a small number of qualified doctors in the Palestinian Ministry of Health facilities	260 (42.7)	101 (50.8)	159 (38.8)	0.005
	Difficulty making an appointment with the doctor	156 (25.6)	60 (30.2)	96 (23.4)	0.08
	Feeling worried about wasting doctor’s time	105 (17.2)	50 (25.1)	55 (13.4)	< 0.001
	Difficulty talking to doctor	101 (16.6)	51 (25.6)	50 (12.2)	< 0.001
Practical	Would try some herbs first	310 (50.9)	116 (58.3)	194 (47.3)	0.012
	Would think that symptom is not something important to see the doctor for	305 (50.1)	102 (51.3)	203 (49.5)	0.73
	Too busy to go to the doctor	304 (49.9)	88 (44.2)	216 (52.7)	0.06
	Has other things to do	288 (47.3)	102 (51.3)	186 (45.4)	0.19
	Financial hardship that will not allow seeing the doctor	188 (30.9)	93 (46.7)	95 (23.2)	< 0.001
	Difficulty arranging transports to the doctor	122 (20.0)	59 (29.6)	63 (15.4)	< 0.001
	Family or partner would refuse the participant being examined by a doctor of a different gender	59 (9.7)	28 (14.1)	31 (7.6)	0.013
	Family or partner would refuse the visit to the doctor	32 (5.3)	19 (9.5)	13 (3.2)	0.002

n = number of participants, WBJ = West Bank and Jerusalem.

All *p*-values are for Pearson’s Chi-square test.

On the multivariable analysis, displaying fair or good awareness of CRC symptoms was associated with a higher likelihood of reporting at least three emotional barriers to early presentation (OR = 1.71, 95% CI: 1.06–2.77 and OR = 1.67, 95% CI: 1.02–2.73, respectively) (Table 6). However, there was no association between CRC symptom awareness level and reporting practical or service-related barriers.

**Table 6:** Multivariable logistic regression analyzing the association between having at least the median number of barriers to seek medical advice for a possible colorectal cancer symptom and participant characteristics.

Awareness of CRC symptoms	All barriers		Emotional barriers		Service barriers		Practical barriers	
	AOR (95% CI)*	*p*-value	AOR (95% CI)*	*p*-value	AOR (95% CI)*	*p*-value	AOR (95% CI)*	*p*-value
Poor	Ref	Ref	Ref	Ref	Ref	Ref	Ref	Ref
Fair	1.35 (0.84- 2.18)	0.21	1.71 (1.06- 2.77)	0.028	0.86 (0.53- 1.40)	0.55	0.80 (0.50- 1.28)	0.35
Good	1.12 (0.69- 1.82)	0.65	1.67 (1.02- 2.73)	0.042	0.87 (0.53- 1.41)	0.56	0.89 (0.54- 1.44)	0.62

AOR = adjusted odds ratio, CI = confidence interval, WBJ = West Bank and Jerusalem, CRC = colorectal cancer.

All analyses were adjusted for age-group, gender, educational level, occupation, monthly income, having a chronic disease, following a vegetarian diet, knowing someone with cancer, marital status, residency, and site of data collection.

The outcome in all models is binary with the median number of barriers in each category utilized as the cutoff.

All *p*-values are for the multivariable logistic regression analysis.

## Discussion

In this study, participants reported that it would take them less time to present with possible CRC signs/symptoms that are related to blood or a mass than other CRC signs/symptoms. Seeking immediate medical advice was most likely to be associated with bleeding from the back passage, and least likely with anemia. Other symptoms displayed a wide variation in reporting immediate help-seeking. Practical barriers were the most frequently reported barriers by study participants. Displaying good awareness of CRC signs/symptoms was associated with a higher likelihood of seeking medical advice within a week from recognizing the sign/symptom and a greater likelihood of reporting emotional barriers.

### Immediate seeking of medical advice for a possible CRC sign/symptom

Bleeding from the back passage was the most commonly reported symptom to seek immediate medical advice (59.5%), which could be explained by a possible tendency of the Palestinian population to associate bleeding with ominous diagnoses. This is supported by findings from a study by Koo and colleagues, who demonstrated that the nature of the presenting signs/symptoms could be a critical influencing factor in the interval of seeking medical advice^30^. In addition, Courtney and colleagues surveyed 1592 participants, aged between 56 and 88 years, and found that 18% of them had experienced rectal bleeding but had never sought medical advice. The reason for this delay was the perception of the respective signs/symptoms as being mild and not serious or as being benign^31^. This could also explain the lower proportions of study participants who reported immediate help-seeking for non-specific CRC signs/symptoms or with gastrointestinal manifestations in this study; as these might also have been perceived as benign symptoms based on the findings and observations from this study. Another possibility could be that some participants may have expected their symptoms to subside over time without the need to visit a doctor, which is consistent with findings of a national study in the United States showing that perception of symptom self-resolution contributed to delay in seeking medical care^32^. Moreover, participants could delay help-seeking due to the hard economic situation in Palestine, especially in the Gaza Strip where about 65% of the population suffer from poverty and 60% are unemployed^33^.

### Association between awareness level of CRC signs/symptoms and seeking medical advice

Mhaidat and colleagues conducted a cross-sectional study in Jordan and reported that 60.6% of participants would seek medical advice within one week of experiencing CRC symptoms. The authors suggested that promoting early help-seeking can be achieved by raising the awareness of CRC signs and symptoms^34^. This study supports their findings, where it demonstrated that good awareness levels of CRC signs/symptoms were associated with a greater likelihood to seek medical advice for any or all of the CRC signs/symptoms within one week. In addition, Niksic and colleagues showed that higher awareness of CRC signs/symptoms led to an increase in the cancer survival index^35^. Moreover, a previous study conducted in Malaysia demonstrated how using mass media campaigns helped to significantly improve the recognition of CRC signs and symptoms^36^. These findings collectively reflect the importance of integrating educational interventions in healthcare and educational systems, which may have a significant impact on patients’ ability to recognize possible CRC signs/symptoms, and thus, increase the likelihood of early help-seeking, which may potentially reduce morbidity and mortality related to CRC^37,38^.

### Barriers to early presentation and association with CRC symptom awareness

A previous study conducted in the Gaza Strip in 2021 reported that emotional barriers to seeking medical advice, in case of having cancer signs/symptoms, were the most commonly reported barriers, where ‘feeling worried about what the doctor might find’ was the most commonly reported barrier to early presentation^8^. The authors also reported the barrier ‘too busy to go to the doctor’ as the most common practical barrier and ‘difficulty making an appointment with the doctor’ as the most common service-related barrier^8^. A previous study from Oman found that lacking time to visit the physician was the most common overall barrier hindering early presentation, followed by emotional barriers^39^. In our study, the most reported barriers to seeking medical advice for possible CRC signs/symptoms were practical, followed by emotional and service-related barriers. This is concordant with findings of a study conducted in New Zealand, showing that patients’ appraisal and normalization of symptoms led to delays in seeking medical advice and therefore, delays in prompt diagnosis of CRC^40^. Furthermore, a study conducted in Jordan showed that 3.1% of participants reported not wanting to visit physicians for their symptoms due to a combination of service, practical, and emotional barriers^41^. This study found that the thought of ‘small number of qualified doctors in the Palestinian Ministry of Health facilities’ was the most common service-related barrier. This is also demonstrated in a study done in New Zealand, which showed that poor perception of physicians led to deterioration in the physician–patient relationship that subsequently acted as a barrier to early presentation and diagnosis of CRC^40^. These findings demonstrate the vital role of patients’ perception of physicians’ technical and interpersonal communication competence in their decision making to seek medical advice, and follow-up appropriately thereafter.

Another important practical barrier reported in this study was thinking that the sign/symptom is not important for the doctor to check. This is consistent with findings by Al-Azri and colleagues who surveyed 999 participants in Oman and found that 21.2% were worried about wasting the physician’s time^39^. Interestingly, another study conducted in Jordan showed that some participants did not want to visit the physician for their possible symptoms without displaying any specific reason explaining such behavior^41^. Moreover, a study from Spain demonstrated that non-specific signs/symptoms (e.g., change in bowel movement) would significantly delay presentation of CRC^42^. This might be due to a lack of awareness and/or faulty perception especially in regards to signs/symptoms recognized as “non-alarming”.

Noticeably, good awareness of CRC signs/symptoms was associated with a higher likelihood of reporting emotional barriers. This could possibly be due to the amplifying effect of fears and concerns after recognizing CRC signs/symptoms, which is consistent with findings reported in a previous study conducted in Lebanon^43^. Educational interventions should help participants to gain insight about the availability of diagnostic and screening modalities, and treatment options, which could potentially have a positive impact on early help-seeking^44,45^. This may mitigate the influence of emotional, practical, and service barriers on individuals’ behavior by helping them understand that early detection of CRC could lead to a significant reduction of the disease burden.

### Future directions

This study explored the anticipated time to seek medical advice and the various barriers that could hinder early presentation of CRC. Using educational interventions that focus on overcoming these barriers and changing the attitudes of the public could possibly shape help-seeking behaviors and lead to earlier seeking of medical advice and, thus, a better overall prognosis. A multidisciplinary and culturally adapted approach should be used to enlighten healthy individuals about CRC, and help them rationalize the fears and false presumptions regarding the disease. Efforts are also needed to address the concerns the public has about the small number of qualified doctors who are working in the facilities of the Palestinian Ministry of Health. Furthermore, qualitative studies are needed to further explore reasons for the delay in help-seeking to understand why these barriers exist.

### Limitations of the study

This study has some limitations that should be considered while interpreting the results. Convenience sampling was utilized to recruit the study participants, which may limit the generalizability of the study findings. However, the large sample size and data collection from various geographical locations in Palestine might have mitigated this limitation. Patients or visitors to oncology departments and those with medical backgrounds were excluded from the study, which might have reduced the number of participants with presumably good CRC symptom awareness. Their exclusion, however, was meant to make this study more pertinent as a measure of the public awareness and perception of CRC. Furthermore, this study sampled participants who did not experience actual CRC symptoms and looked at their perceived knowledge and barriers to early presentation. This may differ from experiencing CRC symptoms and future studies are needed to estimate the time taken by CRC patients to seek medical advice and to examine the interplay between CRC symptom awareness and barriers to early presentation.

## Conclusion

In this study, CRC signs and symptoms with blood or a mass were perceived as the most alarming and prompted earlier help-seeking behavior. Good awareness of CRC signs/symptoms was associated with a higher likelihood of seeking medical advice within a week from recognizing the sign/symptom, but also with a greater likelihood of reporting emotional barriers. The most frequently reported barriers to early presentation were practical barriers. Establishing educational interventions to raise CRC awareness may help in promoting earlier help-seeking and, thus, facilitate earlier diagnosis and improved prognosis.

## Data Availability

The dataset used during the current study is available from the corresponding author on reasonable request.
